# Constraining the response of continental-scale groundwater flow to climate change

**DOI:** 10.1038/s41598-022-08384-w

**Published:** 2022-03-16

**Authors:** Ben Mather, R. Dietmar Müller, Craig O’Neill, Adam Beall, R. Willem Vervoort, Louis Moresi

**Affiliations:** 1 EarthByte Group, School of Geosciences, The University of Sydney, Camperdown, NSW 2006 Australia; 2 Sydney Informatics Hub, The University of Sydney, Darlington, NSW 2008 Australia; 3 Department of Earth and Environmental Science, Macquarie University, Sydney, NSW 2109 Australia; 4 School of Earth and Environmental Sciences, Cardiff University, Cardiff, CF10 3AT Wales, UK; 5 School of Earth, Atmospheric and Environment, Monash University, Melbourne, Victoria 3800 Australia; 6 ARC Training Centre Data Analytics for Resources and the Environment (DARE) and School of Life and Environmental Sciences, The University of Sydney, Camperdown, NSW 2006 Australia; 7 Research School of Earth Science, Australian National University, Canberra, ACT 0200 Australia

**Keywords:** Solid Earth sciences, Geodynamics, Hydrogeology

## Abstract

Numerical models of groundwater flow play a critical role for water management scenarios under climate extremes. Large-scale models play a key role in determining long range flow pathways from continental interiors to the oceans, yet struggle to simulate the local flow patterns offered by small-scale models. We have developed a highly scalable numerical framework to model continental groundwater flow which capture the intricate flow pathways between deep aquifers and the near-surface. The coupled thermal-hydraulic basin structure is inferred from hydraulic head measurements, recharge estimates from geochemical proxies, and borehole temperature data using a Bayesian framework. We use it to model the deep groundwater flow beneath the Sydney–Gunnedah–Bowen Basin, part of Australia’s largest aquifer system. Coastal aquifers have flow rates of up to 0.3 m/day, and a corresponding groundwater residence time of just 2,000 years. In contrast, our model predicts slow flow rates of 0.005 m/day for inland aquifers, resulting in a groundwater residence time of $\sim$ 400,000 years. Perturbing the model to account for a drop in borehole water levels since 2000, we find that lengthened inland flow pathways depart significantly from pre-2000 streamlines as groundwater is drawn further from recharge zones in a drying climate. Our results illustrate that progressively increasing water extraction from inland aquifers may permanently alter long-range flow pathways. Our open-source modelling approach can be extended to any basin and may help inform policies on the sustainable management of groundwater.

## Introduction

The velocity field of continent-scale groundwater flow is broadly characterised by recharge in regions of high topography, where the water table is elevated, and discharge from continental slopes into the ocean^1^. While this approximation does not hold at smaller scales where spatial variations in the permeability due to structural and lithological inhomogeneities control the flow pathways^2^, models which encompass multi-scale groundwater flow patterns offer an opportunity to understand the hydrologic cycle under a changing climate^3^. Most continent to global-scale groundwater studies have focused on the variations of water table depth^4^, shallow groundwater modelling in unconfined aquifers^5,6^, or regional flow patterns subject to climate forcing^7^. Combining observations with models to predict groundwater sustainability in response to climate change is a global challenge^8^, and data-driven continental-scale models which encompass deep aquifers controlled by geology represents a particular gap in knowledge. Such models present a significant computational challenge to operate at sufficient resolution honouring deep geometrical and hydraulic structures, particularly where it is necessary to run ensembles to explore uncertainty of the hydraulic regime. We present a numerical framework for coupled hydro-thermal modelling to resolve the flow dynamics within deep aquifers of continental-scale basins. The framework is designed for efficient parallel scaling on high performance computing (HPC) infrastructure and has been built to integrate common hydraulic observations, including hydraulic head measurements in boreholes and recharge estimates from the chloride mass balance method^9^. Data assimilation uses a Bayesian framework, where the hydraulic conductivity of each geological layer in the basin is inverted to an optimal value consistent with the constraining data. The inverse numerical framework we have built is general and can be applied to basins worldwide to quantify depth-dependent groundwater flow rates and directions, submarine groundwater discharge and saline marine water ingress into coastal aquifers. It provides constraints on the degree of mixing between deep aquifers and alluvial sediments, and can explore the re-calibration of flow pathways under different groundwater pumping scenarios. Multi-scale, whole-of-basin approaches to modelling can resolve the larger-scale effects and changes that underpin the groundwater resources used for agricultural production, mining, industry, and urban use.

In this paper we apply our numerical framework to the Sydney–Gunnedah–Bowen (SGB) Basin in eastern Australia. The SGB Basin spans approximately 1.5 million square kilometres, which we model at exceptionally high resolution in 3D (over 10 million cells or $6 \times 6 \times 0.05$ km resolution in the *x*, *y*, *z* directions, respectively) to resolve the regional flow patterns down to 12 km beneath the crust. These models reveal the long range feedback between recharge, transport, and discharge between regional aquifers and provide a context for small-scale, ultra high resolution models. Our models are constrained from borehole observations of hydraulic head, groundwater recharge from the chloride mass balance method, and temperature gradients.

### Motivation for groundwater modelling in eastern Australia

**Figure 1:**
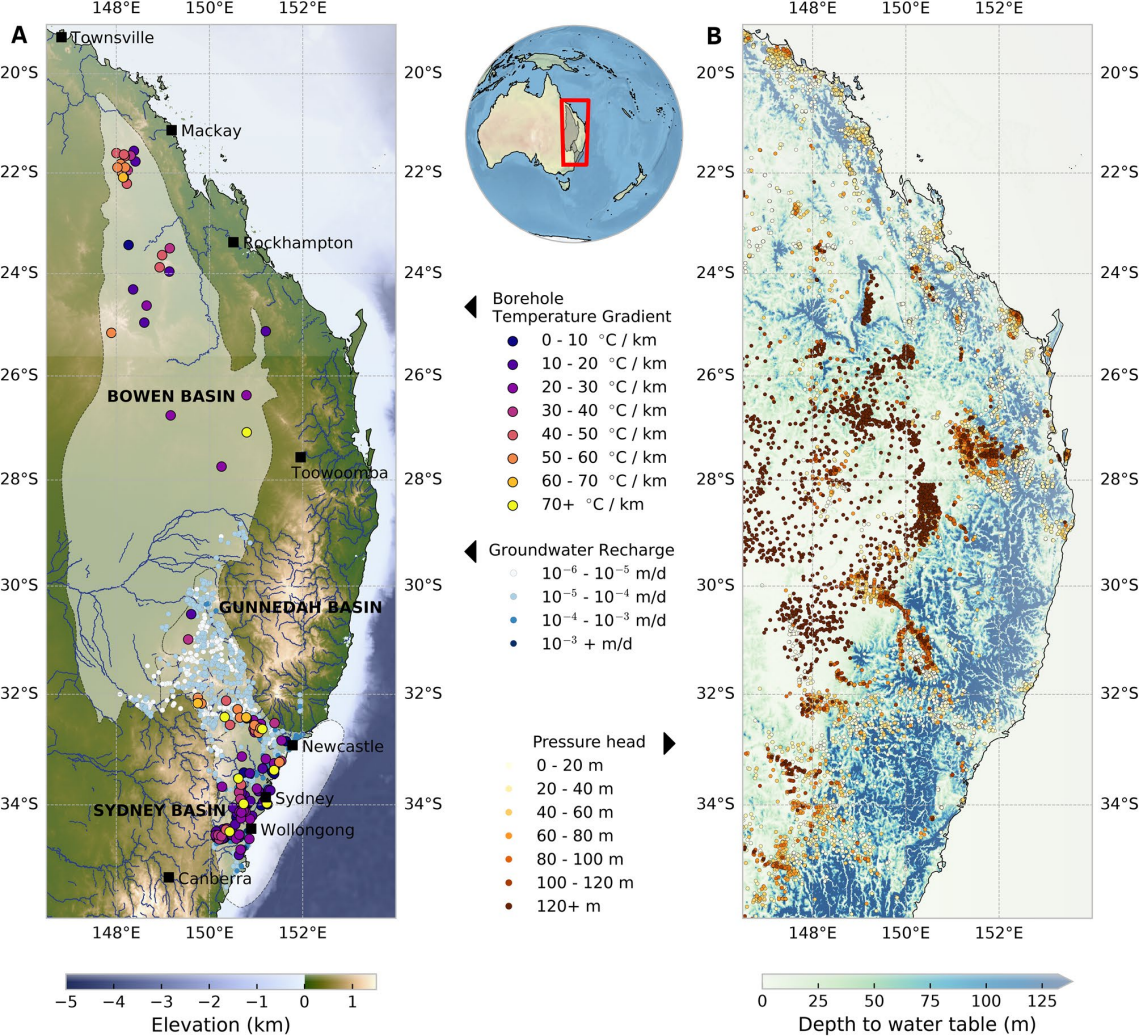
The boundary of our model covers much of the eastern third of Australia with the Sydney–Gunnedah–Bowen Basin highlighted in white. Multiple data types are used to constrain our coupled groundwater-heatflow models, including maps of (**A**) elevation of the study area overlain with borehole measurements of temperature gradients^19^ and groundwater recharge rates^9^. Blue lines indicate major perennial streams. (**B**) The depth to the water overlain with the hydraulic pressure head ($h_P = P/\rho _w g$ in metres) measured in groundwater boreholes (Table S1). Satellite imagery was obtained from the ETOPO1 global relief model^20^.

Groundwater is a crucial resource in Australia, the world’s driest inhabited continent. Increasing aridity and agricultural water-utilisation have placed progressive strain on Australia’s inland water resources, and Australia’s reliance on groundwater is expected to increase due to population growth and a higher propensity for drought^10^. Changes in groundwater depth are known to affect the susceptibility of regions to changes in temperature and precipitation^11^, which is compounded by increased evapotranspiration due to a warmer climate and, in-turn, a diminished capacity for groundwater storage^12^. While an administratively strict licensing system exists for groundwater use in Australia, this relies on the limited knowledge about the capacities and flow rates in aquifers interpreted from spatially sparse borehole data across a large region^10,13^. The Sydney-Gunnedah-Bowen (SGB) Basin comprises part of the Great Artesian Basin, the largest and deepest artesian basin in the world, and supports agricultural production, mining, industry and urban activities across much of the eastern third of the continent (Fig. 1). Regional groundwater levels have declined since 2000 due to pumping of shallow alluvial sediments^10^, yet the rate of extraction to maintain environmental flows is unknown despite the risk of permanently reducing groundwater storage^14^, and increasing groundwater and river salinity from redirected flow pathways^15^. Rates of groundwater flow have previously been measured using isotopic tracers obtained from shallow boreholes^16^, and recharge rates have been inferred from the concentration of chloride in rainfall and groundwater^9^ to estimate local flow rates in specific areas of the SGB Basin. However, the flow dynamics within deep aquifers is largely unconstrained and the degree to which deep aquifers recharge the more productive and higher quality shallow alluvial sediments at the basin scale is relatively unexplored. Existing workflows used to model groundwater assume topography-driven groundwater flow from regions of high to low elevation^17^, but they struggle to capture the intricate flow pathways between deeper aquifers and the surface within continental-scale basins such as the SGB Basin. Our inverse modelling framework accomplishes this by making the following advances: (i) solving the equations that govern groundwater flow using a finite-element particle-in-cell method on deformed meshes^18^, (ii) resolving multi-scale flow patterns and communication between deep and near-surface aquifers over continent-scale domains, (iii) casting the inversion of hydraulic properties of aquifers using a Bayesian approach.

**Figure 2:**
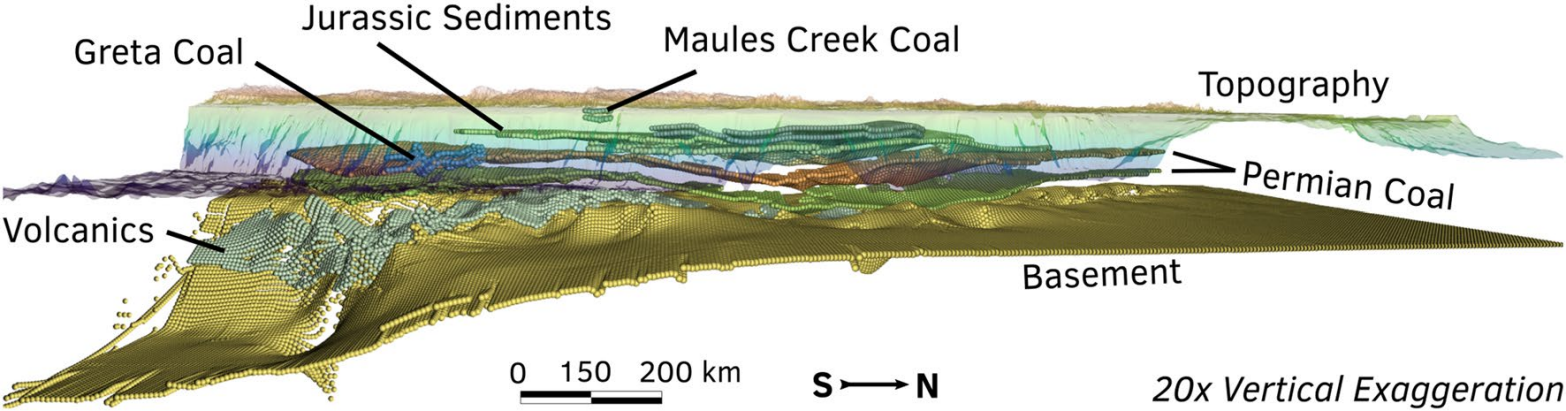
3D stratigraphy of the Sydney–Gunnedah–Bowen Basin. The vertical spacing of layers has been exaggerated for visual clarity. The model of the basin was rendered in 3D using Underworld^18^.

**Table 1:** Material properties assigned to major lithologies in the Sydney–Gunnedah–Bowen Basin. Bracketed numbers indicate one standard deviation from the mean—i.e. mean (std). Units are $\rho$ = density (kg m$^{-3}$), $k_T$ = thermal conductivity (W m$^{-1}$K$^{-1}$), $k_h$ = hydraulic conductivity (m s$^{-1}$), *H* = rate of heat production (W m$^{-3}$).

Lithology	$\rho$	$k_T$	$H \times 10^{-6}$	$\log _{10} k_h$
Maules Creek Coal	1900	0.3 (0.1)	0.0 (0.1)	$-$7 (1)
Jurassic coal	2180	0.3 (0.1)	0.0 (0.1)	$-$7 (1)
Jurassic sediments	2310	2.0 (0.5)	1.2 (0.5)	$-$6 (1)
Greta coal	1900	0.3 (0.1)	0.0 (0.1)	$-$7 (1)
Permian Coal Measures	2180	0.3 (0.1)	0.0 (0.1)	$-$7 (1)
Reid Dome Beds	2540	2.0 (0.5)	1.2 (0.5)	$-$6 (1)
Permian sediments	2370	2.0 (0.5)	1.2 (0.5)	$-$6 (1)
Denison Volcanics	2950	2.8 (0.5)	0.5 (0.2)	$-$8 (1)
Late Carboniferous Volcanics	2950	2.8 (0.5)	0.5 (0.2)	$-$8 (1)
Basement	2650	3.0 (0.8)	2.0 (0.5)	$-$10 (1)

### Regional groundwater dynamics in eastern Australia

The bulk flow field of the SGB system is largely fed by recharge from the Great Dividing Range and discharged out to the Tasman Sea with previously estimated residence times ranging from < 70 years to > 100 000 years^16^. Smaller topographic variations overprint the regional flow pattern. Flow rates are generally determined by the hydraulic conductivity of sedimentary layers within the basin. Permeable rocks, such as Jurassic sediments, are efficient aquifers, whereas rift volcanics deposited during the Early Permian generally behave as aquitards that limit the flow of water. It has long been recognised that shallow alluvial sediments are partly recharged by leakage from deeper aquifers of the SGB Basin, which comprise the eastern portion of the Great Artesian Basin^16,21^, although the scale of this feedback is poorly understood. Through integrating data with a geological model of basin architecture, we are able to probe the complex fluid pathways from recharge to discharge and the degree of mixing between each layer.

The SGB Basin architecture is described here by a deep 3D model constrained by gravity modelling and lithology data from core samples^22,23^. The region contains abundant Permian–Jurassic black coal measures and mafic volcanic material interspersed with sandstone and mudstone layers. We populate a 3D model of basin architecture (Fig. 2) with initial thermal and hydraulic material properties described in Table 1. These starting values of lithology-dependent thermal and hydraulic parameters are reference values obtained from material averages in the literature^24–26^ and form the *a priori* information used within the inversion (refer to “Methods” section). The 3D stratigraphic model does not resolve the individual formation-scale rock units of the Sydney Basin, but condenses these formations into the dominant lithologies that adequately describe the geological heterogeneity across large regions. The thermal and hydrological properties that are mapped to layers in the 3D model are adjusted by temperature-dependent and depth-dependent relationships within our numerical modelling workflow (refer to “Methods” for a detailed description of the governing equations).

## Data assimilation

Each borehole facilitates an independent observation of the hydro-thermal field which jointly provides valuable constraints on subsurface temperature and the groundwater flow field in our coupled system. Our approach here is to cast the inverse problem of coupled heat conduction and groundwater flow within a Bayesian framework, which inverts lithology-dependent hydraulic conductivity ($k_h$), thermal conductivity ($k_T$), rate of heat production (*H*), and the lower temperature boundary condition ($T_1$) from borehole data (refer to the “Methods” section for a detailed description of the inverse problem.) This then allows us to develop a coupled basin-scale thermal and groundwater model that satisfies (i) hydraulic head measurements, (ii) groundwater recharge data, and (iii) borehole temperature data, and use this to explore large-scale groundwater dynamics of the SGB Basin.

### Hydraulic head measurements

**Figure 3:**
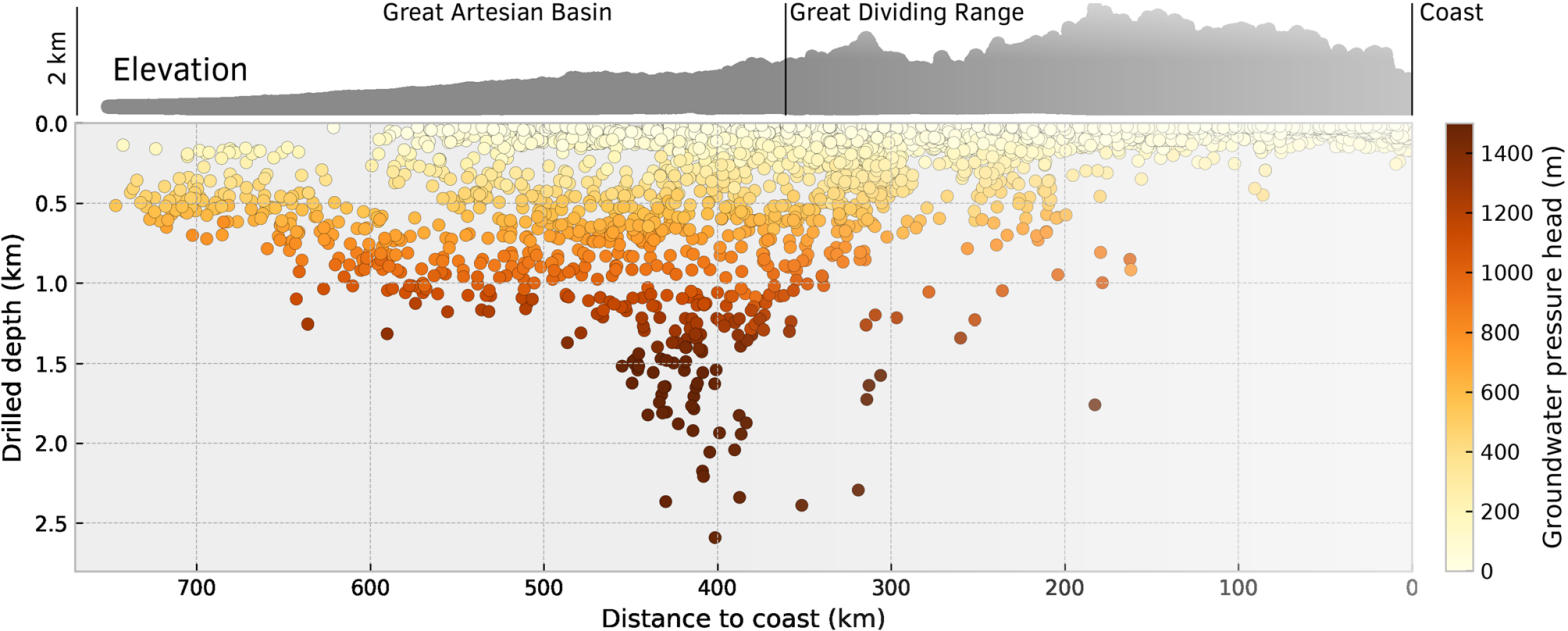
Mean hydraulic pressure head ($h_P = P/\rho _w g$) in 15,018 groundwater monitoring boreholes within the SGB Basin recorded before the year 2000. The hydraulic pressure head is visualised in favour of the hydraulic head to aid the interpret the variation of aquifer pressure independent of topography. These data are collapsed from Fig. 1B to illustrate the lateral variation in pressure head across the basin as a function of drilled depth. The highest pressures are found in deep boreholes inland from the Great Dividing range.

We use the database of groundwater level monitoring sites from the National Groundwater Information System (NGIS, http://www.bom.gov.au/water/groundwater/ngis/) to constrain hydraulic head within major aquifers of the SGB Basin. 15,018 monitoring boreholes have been extracted from the NGIS and filtered to include groundwater level observations prior to the year 2000 (Table S1). This study is primarily focused on natural groundwater flow rates, thus we exclude water levels measurements that were made after 2000 where groundwater extraction may have increasingly affected the water pressures in aquifers^13^. The spatial distribution of observation boreholes is spread relatively uniformly across the study area (Fig. 1). 90% of inland boreholes penetrate less than 150 m deep; the remainder extend down to 2500 m predominantly in inland regions and intersect the confined aquifers of the Great Artesian Basin (Fig. 3). As such, these bores have very high pressure heads^13,27^. The integration of hydraulic head measurements within our inverse modelling framework constrains the hydraulic conductivity of major lithologies. In particular, high pressures observed within inland aquifers must be maintained by the low-permeability of overlying lithologies.

### Groundwater recharge data

Groundwater recharge is difficult to quantify as it cannot be directly measured. Instead, geochemical proxies can be used to constrain recharge rates such as the chloride mass balance method^28,29^. Chloride is excluded from evapo-transpiration; thus, assuming no change in salt storage over very long time periods, recharge, $q_r$, can be estimated from the rate of precipitation and the ratio of chloride concentration in rainfall, $Cl_{p}$, and groundwater, $Cl_{gw}$,

1 $$\begin{aligned} q_r = \frac{q_p Cl_{p}}{Cl_{gw}} \end{aligned}$$

The mass of Cl into the groundwater system from precipitation, $q_p$, is balanced by the drainage out of the system after budgeting for Cl lost to surface runoff. Equation (1) can be expanded to consider the lateral flow of chloride with surface water runoff,

2 $$\begin{aligned} q_r = \frac{q_p Cl_{p} + [q_{\mathrm {on}}Cl_{\mathrm {on}} - q_{\mathrm {off}}Cl_{\mathrm {off}}]}{Cl_{gw}} \end{aligned}$$

where $Cl_{\mathrm {on}}$ and $Cl_{\mathrm {off}}$ is the concentration of chloride in runon and runoff, respectively. The chloride mass balance method assumes that Cl is conservative within the system and there is no variation in chloride fluxes through time^9,30^. We incorporate 1279 recharge estimates from Cl measurements in boreholes to constrain the recharge rates of shallow rock units within SGB Basin (Table S2)^9^. These measurements are mostly clustered within the Sydney and Gunnedah portions of the SGB Basin system. The high variability of recharge rates (0.001–2 mm/day) can be attributed to heterogeneous surface geology, which have hydraulic conductivities that differ by orders of magnitude (Table 1). Cl fluxes provide valuable constraints for recharge rates within the basin. By assimilating these recharge data across the entire basin, with their respective uncertainties, we can constrain the hydraulic conductivity of major aquifers and estimate groundwater flow rates.

### Borehole temperature data

**Figure 4:**
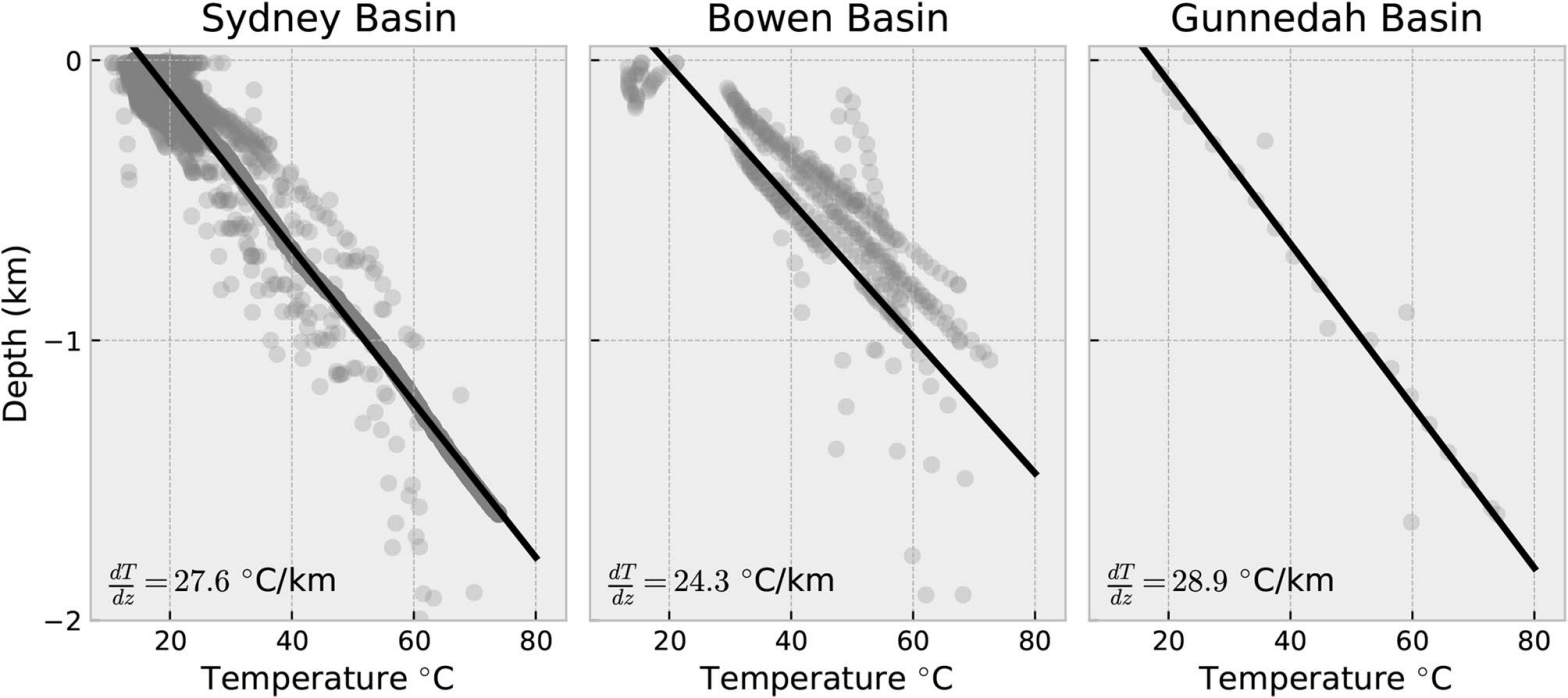
Temperature-depth profiles across Sydney–Gunnedah–Bowen Basin down to 1 km depth. Temperature gradients vary considerably within each sub-basin but average to 27.6, 24.3, 28.9 $^{\circ }$C/km for the Sydney, Bowen, and Gunnedah Basin, respectively.

We use borehole temperature data to indirectly estimate groundwater flow velocities from their perturbation of temperature gradients. These extend up to 3 km deep, which resolves deeper long-range flow velocities than can be observed from groundwater monitoring boreholes that rarely extend below 300 m. This is possible because upward and downward groundwater flow results in lower and higher geothermal gradients, respectively, which are observed where aquifers and boreholes intersect^31,32^. Depending on the temperature differential and the hydraulic conductivity of an aquifer, thermal perturbations measured within boreholes may pervade a large region^33^. Temperature gradients vary considerably within the SGB Basin (Fig. 4). The average temperature gradient within 268 boreholes is approximately 26 $^{\circ }$C/km and most of the data are clustered within the Sydney and Gunnedah Basins^19^ (Table S3). Some are derived from industry data that have been filtered for unequilibrated bottom-hole temperature measurements. Palaeoclimate corrections have been applied to each borehole consistent with the surface temperature history of the region^19^.

## Results

**Figure 5:**
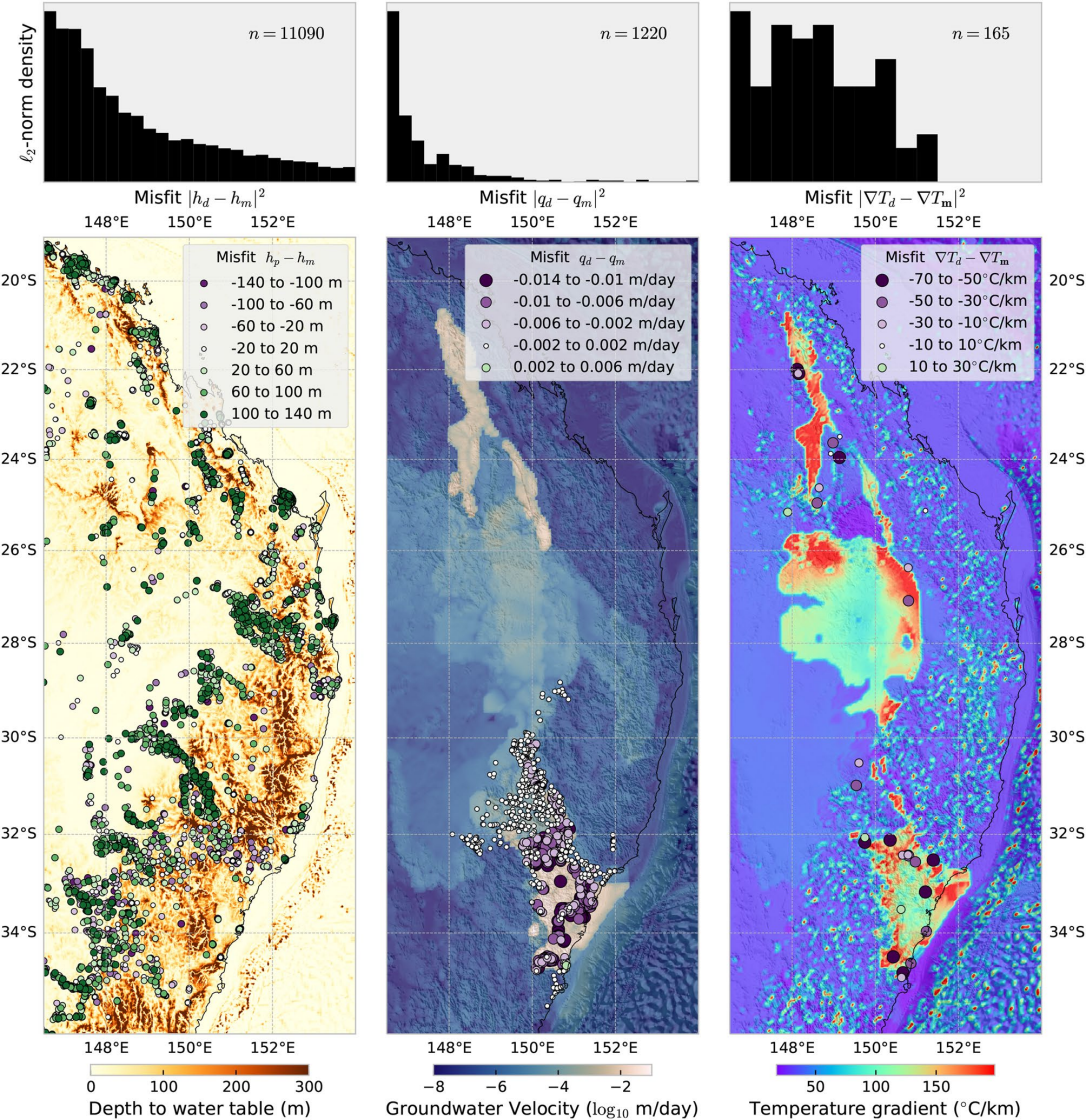
Map of misfit between simulated groundwater pressure, velocity and temperature gradients (from the MAP estimate) with borehole observations. The $\ell _2$-norm misfit histograms indicate the optimal fit between model and data. These maps were generated from our models using Cartopy 0.19 (https://scitools.org.uk/cartopy).

We identified the optimal model of groundwater flow and thermal structure from our ensemble as the model that obtains the best fit between available hydrologic and thermal data with prior information (Table 1). This model, the maximum *a posteriori* (MAP) model, of lithology-dependent hydraulic conductivity, thermal conductivity, rate of heat production, and lower temperature boundary condition was found after 6600 evaluations of the forward model using the inversion approach outlined in the “Methods” section. We obtain identical MAP estimates if we increase the number of seed points for the optimisation algorithm^34^, which suggests we have identified the global minimum of the objective function (“Methods” section). For the most part, we are able to reproduce the high degree of variability between our model and the data (Fig. 5). The model misfits (measured as $\ell _2$-norm) follow a logarithmic decay for each dataset with the exception of the temperature gradients. These are skewed towards zero, but do not decay logarithmically with increasing misfit because (i) there are relatively few measurements ($n=165$), (ii) much of the data is very shallow and are taken within interbedded coal-sand layers, and (iii) temperature gradients can vary considerably depending on circulation time after drilling^19^. Groundwater recharge velocities are heavily skewed towards zero misfit within the Sydney Basin, and larger misfits proximal to the coast. Smaller misfits observed within the highlands indicate that the recharge of groundwater into deeper aquifers of the SGB Basin are concordant with observations, whereas coastal outflow points are of lesser concern towards the very end of their flow pathways out to the ocean. Conversely, low misfits between our simulation and the hydraulic head observed in groundwater monitoring boreholes is more important for inland regions where much of the agricultural production is located, rather than highland regions. Seasonal rainfall exerts a much stronger influence on variation in water levels observed at recharge points, which helps to explain the larger misfits of hydraulic head along the eastern highlands.

**Figure 6:**
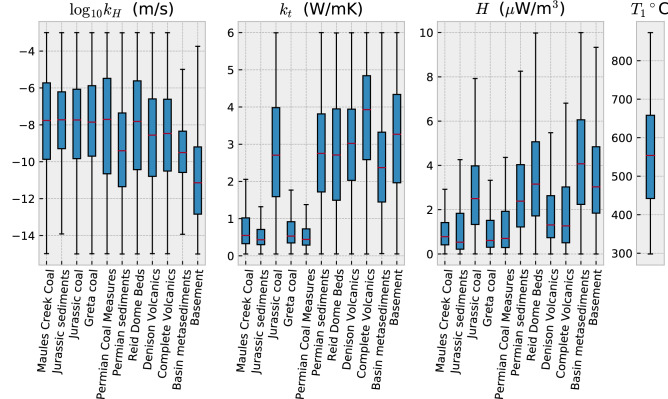
MAP estimates for lithology-dependent values of hydraulic conductivity ($k_H$), thermal conductivity ($k_t$), rates of heat production (*H*), and the lower temperature boundary condition ($T_1$). Results are presented as box plots where whiskers indicate the total spread of values, blue boxes indicate the lower and upper quartiles with a red line at the median.

The posterior probability distribution of each parameter is given in Fig. 6. The lower temperature boundary condition, $T_1$, is most sensitive to the temperature data: from a uniform prior distribution between 100–900 $^{\circ }$C, we obtain $T_1 = 521 \pm 98$ $^{\circ }$C *a posteriori*. The values of lithology-dependent hydraulic conductivity are mostly constrained from groundwater recharge rates we assimilated from Cl concentrations^9^, and hydraulic head measurements in NGIS groundwater monitoring bores. Lithologies with the largest volume in our model are the most constrained since they have a proportionally larger impact on groundwater flow velocities and water levels observed at monitoring sites. Furthermore, the proportion of hydraulic head measurements within lithologies of higher permeability is considerably greater than other lithologies. The Jurassic sediments, basin metasediments, and Late Carboniferous volcanic layers are best constrained by the data.

**Figure 7:**
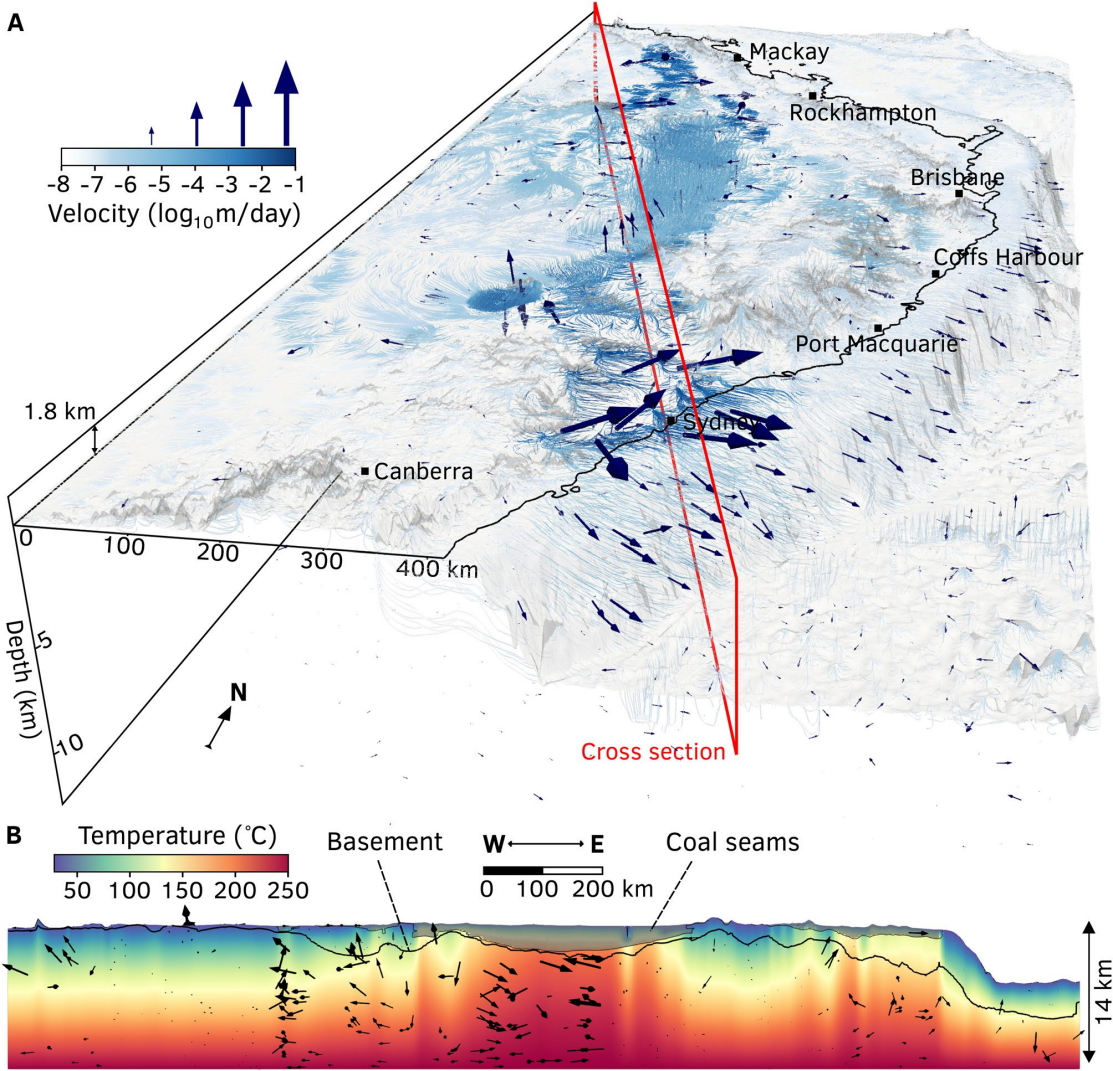
Coupled heat-groundwater flow model of the Sydney–Gunnedah–Bowen Basin based on the MAP estimate of material properties and boundary conditions. (**A**) Groundwater velocity field with coal seams outlined in grey overlain with temperature gradients measured in boreholes. This visualisation of the velocity field obtained from our model was rendered in 3D using Paraview 5.9 (https://www.paraview.org). (**B**) temperature field overlain with heat flux vectors. The 2D slice was generated from our models using Matplotlib 3.4 (https://matplotlib.org).

**Figure 8:**
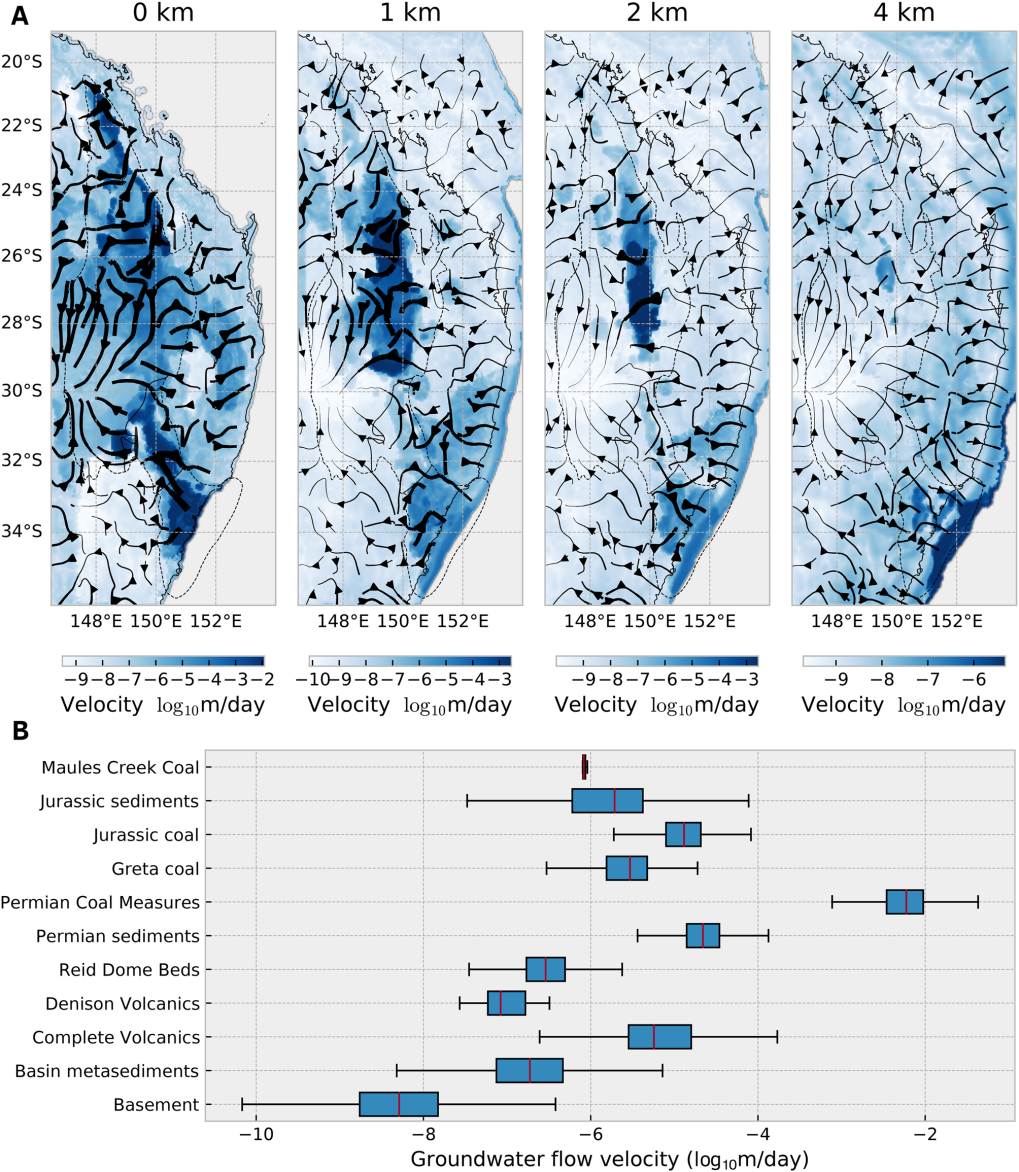
(**A**) Depth slices through the Sydney-Gunnedah-Bowen Basin showing the groundwater flow velocity in $\log _{10}$ m/day. Streamlines indicate the direction of horizontal flow, their thicknesses are proportional to the magnitude of velocity. The dashed outline indicates the spatial extent of the SGB Basin. This map was generated from our models using Cartopy 0.19 (https://scitools.org.uk/cartopy). (**B**) Box plot of the groundwater flow velocities through various layers within the model. Due to the depth-dependent hydraulic conductivity, there is generally a reduction of groundwater flow velocities at greater depths.

From the MAP estimate, we obtained the simulated temperature, fluid pressure head, and velocity fields for the SGB Basin (Fig. 7). Flow rates reach a peak of 0.3 m/day at the discharge zone of the Sydney Basin along the continental shelf (Fig. 7A). Depth slices through the basin highlight the carrying ability of the aquifer network to transport groundwater from recharge zones in nearby mountain ranges and also long-range flow from inland regions (Fig. 8). The mountain ranges broadly delineate coastal aquifers from inland aquifers that possess much slower flow rates around 0.005 m/day. These inland aquifers are 1–2 km deep which underlie alluvial sediments which incur the most groundwater pumping. The average distance between recharge and discharge zones in inland aquifers is 700 km, which corresponds to a groundwater residence time of approximately 400,000 years. Contrast this with coastal aquifers, where the average transport distance of an aquifer is 200 km and seepage velocity of 0.3 m/day equates to a groundwater residence time of just 2,000 years. The disconnect between recharge inputs and potential extraction is a significant feature that can only be captured using large-scale basin modelling and is important for groundwater management.

Numerical simulations of flow and heat transport in confined and unconfined carbonates in Hungary reveals the dominance of topography-driven flow in redistributing heat^35^. Here, the coal measures within the Sydney–Gunnedah–Bowen Basin are very effective thermal insulators (0.3 ± 0.1 W m$^{-1}$K$^{-1}$), which refract heat toward more thermally conductive sedimentary layers (Figure 7B). Exceptionally high heat flow values are modelled around the rim of coal seams (100 mW m$^{-2}$), which is comparable to high heat flow measured in Proterozoic regions enriched in high heat-producing elements^36^ or Phanerozoic regions associated with recent volcanism ($\le$ 5 Ma)^37^. These results significantly improve the modelling of 3D heat-flow paths that is consistent across the entire basin. Earlier studies of the regional thermal regime, which primarily relied on forward modelling 2D cross sections to borehole temperature data^19,33^ or downward extrapolation of bottom-hole temperatures to 5 km depth used in geothermal prospectivity maps^38^. The groundwater flow field augments the conductive regime by transporting heat from coal seams toward discharge zones on either side of the mountain range. By performing coupled thermal-hydraulic inversions, our model encapsulates more data that are sensitive to temperature variation than what can be gleaned from heat flow data alone.

## Discussion

The modelling approach we have taken applies large scale numerical modelling of coupled groundwater-heat flow to the SGB Basin using a Bayesian inverse framework. Our models jointly invert the optimal configuration of hydrologic and thermal parameters that best fit the data. The model we have developed can be used to probe the steady-state dynamics of groundwater flow within the SGB Basin and can be a reference point for future time-dependent models that vary groundwater extraction and recharge rates. The framework we have developed may be extended to other regions with little difficulty.

From our MAP model, a difference of three orders of magnitude between coastal and inland aquifer residence times provides valuable insights into sustainable planning of water extraction^27^. Coastal regions are supplied with abundant groundwater due to high recharge from the eastern highlands of Australia. Discharge from the continental shelf into the Pacific Ocean occur at a maximum rate of 0.3 m/day from the Sydney Basin aquifer system. Due to this high rate of discharge and close proximity to recharge zones, it is less prevalent for saline marine water to contaminate coastal aquifers observed elsewhere in the world^39,40^, with only a small number of coastal extraction sites in Newcastle and Queensland reporting saline intrusion^41^. In comparison, flow velocities within inland deep aquifers are between 10$^{-5}$–10$^{-3}$ m /day and have a residence times of approximately 400,000 years. Near-surface alluvial aquifers not modelled here may have higher flow rates and be a more viable groundwater resource, but are also susceptible to seasonal variability in recharge rates and potentially complex interaction with deeper aquifers. The flow within the deep aquifers in our model provides an important constraint for understanding leakage and upward pressure.

### Anthropogenic groundwater extraction

**Figure 9:**
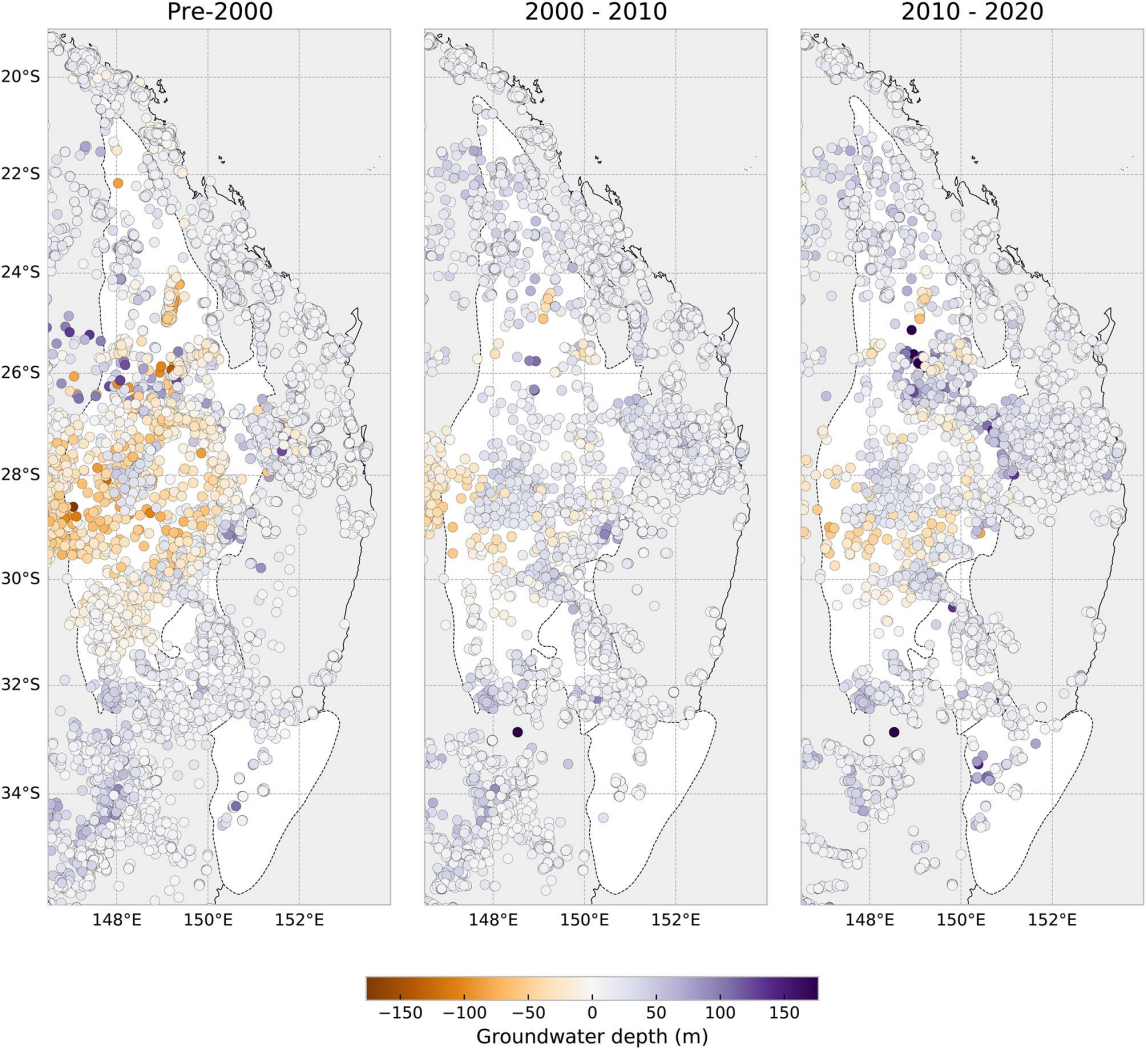
Change in water depth measured in groundwater boreholes pre-2000 to 2020 (negative values indicate artesian pressures translated to meters above the ground surface). These maps were generated from our models using Cartopy 0.19 (https://scitools.org.uk/cartopy).

**Figure 10:**
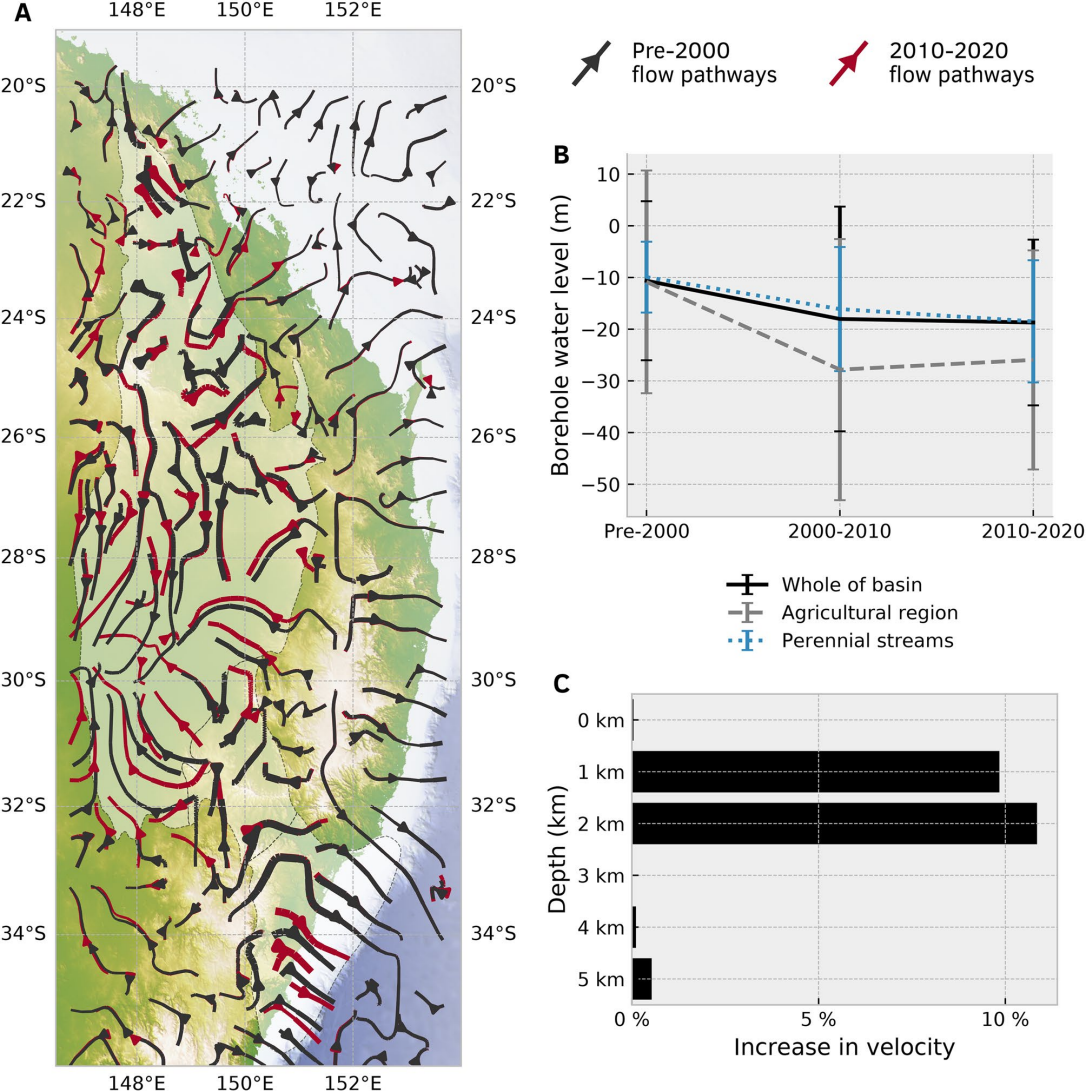
Comparison between regional flow patterns using water table data from pre-2000 to 2010–2020. (**A**) streamlines illustrate the change in regional flow pathways from pre-2000 (dark grey) to 2010-2020 (dark red), where the thickness of each streamline indicates the relative velocity. This map was generated from our models using Cartopy 0.19 (https://scitools.org.uk/cartopy). (**B**) Decline in water levels measured in groundwater boreholes from pre-2000 to 2020. The “agricultural region” is the western portion of the model that comprises part of the Great Artesian Basin (west of 150$^{\circ }$ longitude between 25–32$^{\circ }$S). (**C**) Flow velocities have increased on average by 10% between 1–2 km depth, which indicates that deeper aquifers of the GAB are recharging alluvial sediments within inland regions to replenish water that has been pumped since 2000.

These groundwater flow models infer steady-state fluid velocities from hydraulic data collected before groundwater extraction began to increase significantly post-2000^13,27^. By assimilating data averaged over a long timescale up to 2000, the model we have presented is as close to the natural groundwater dynamics as is possible from the data we have available. In more recent years the rate of extraction has increased throughout the basin^42^, particularly due to the millenium drought, and the water depth measured in groundwater bores have departed from pre-2000 levels (Fig. 9). On average, the entire basin has experienced a 7 m decline in water levels and up to 17 m in agricultural regions west of the highlands (Fig. 10B). Borehole water levels have plateaued in the years since 2010 and if this reaches a new steady state, the storage capacity of near-surface aquifers will have decreased by 4,000,000 GL over the entire model domain. A *La Niña* event between 2010–2011 brought considerably more precipitation to Eastern Australia which may have increased the decadal average between 2010 and 2020, so the reduction in storage capacity of these aquifers may be even further reduced from our estimates.

Re-computing the simulation using a reduction in water table consistent with the change in groundwater levels over 2010–2020 (Table S4) significantly alters the flow patterns from our previous model inferred from pre-2000 water level data (Table S1). The local draw-down of the water table increases the hydraulic head gradient with underlying aquifers, and enhances recharge to the alluvial sediments. Dark red streamlines in Fig. 10A, which represent steady-state flow patterns between 2010 and 2020, reveal a significant departure from pre-2000 streamlines (in dark grey). The fluid pathways are longer as groundwater is drawn further from recharge zones to replenish alluvial sediments that have been pumped since 2000. This is particularly prevalent in inland regions where permeable interbedded Permian sediment and coal layers recharge Jurassic sediments, which in turn recharge alluvial sediments. Isotopic tracers indicate a mixing of groundwaters with residence times of < 70 years and hundreds of thousands of years old, which is consistent with that of the Great Artesian Basin^16^. In keeping with the trace isotope inter-connectivity of alluvial sediments in inland regions, such as the Namoi Valley^21^, with deeper aquifers that comprise the SGB Basin and Great Artesian Basin, our modelling is consistent with the hypothesis that shallow aquifers are recharged by leakage from deeper aquifers.

### Changing groundwater flow pathways

Comparing the variation in velocity along inland flow pathways reveals that aquifers between 1–2 km depth experience 20% higher velocities as a result of groundwater extraction since 2000 (Fig. 10C). These higher velocities are a consequence of deeper aquifers of the SGB and Great Artesian Basin recharging alluvial sediments in certain parts of the SGB where significant pumping has occurred since 2000. The velocity variation at the surface is not uniform: highland regions generally experience a 0–30% increase in flow rates as deeper aquifers are recharged; in contrast some regions that were discharge zones now divert groundwater further west of the highlands by up to 60% of pre-2000 flow rates. The implications of this are two-fold: first, the reduced water table encourages recharge from rivers and lakes into surrounding aquifers, thereby reducing surface water supplies^43^. We find that the water table beneath major perennial streams deepened by an average of 7 m since 2000 (Fig. 10B), which can increase recharge from increasingly drier surface water resources on the western side of the Australian highlands^44^. Second, the diversion of flow pathways from perennial streams since 2000 has important consequences for worsening salinity conditions in upstream catchments and abandoned discharge zones during drought conditions^15^. Increasing groundwater and river salinity directly can directly affect agricultural production in inland regions, particularly where established groundwater flow pathways have been disconnected as a result of pumping. The consequences of groundwater pumping within coastal aquifers is relatively diminished due to higher annual rainfall and lower groundwater residence times. Our simulation assumes that the water table is in equilibrium with recharge from rainfall and extraction from groundwater bores; however, groundwater flow through the alluvial sediments occurs over much shorter timescales than deeper aquifers and will require time-dependent modelling for future extraction and climate scenarios. The long residence time of water within deep aquifers mitigate yearly fluctuations in recharge from precipitation, nonetheless careful management of groundwater resources is important for the continuing groundwater extraction as the Australian continent becomes drier.

Future studies could use these models to inform time-dependent modelling of groundwater flow in response to mountain building and denudation. The long residence time of deep groundwater (400,000 years) is comparable to timescales of tectonic uplift which may, in turn, be influenced by longer-timescale tectonics. Additionally, tectonics controls (i) sediment flux and compaction, and (ii) deep stress, which impacts on the porosity and permeability structure. This would shed light on groundwater dynamics in the wider context of tectonic uplift and erosion along the eastern seaboard of Australia.

## Conclusions

Large-scale groundwater modelling is important to understand the long range flow dynamics of continent-scale basin systems that underpins many of the more productive shallow systems. Here we developed a general numerical framework for modelling deep groundwater flow that can be applied to basins worldwide. Using a geological model of the SGB Basin, we are able to invert flow rates from borehole temperature data to quantify the groundwater residence times at different depths throughout the region. Groundwater flow in the SGB Basin is partitioned into two systems delineated by the eastern highlands of Australia. The relatively flat terrain on the inland side of the eastern highlands drives fluid through inland aquifers at a slow rate of 0.005 m/day. Integrating these flow rates across much of the inland region yields an average residence time of approximately 400,000 years. In contrast, coastal aquifers experience high flow rates up to 0.3 m/day from the Sydney Basin and an average groundwater residence time of 2000 years. Whilst seepage of seawater into coastal aquifers is significant for some parts of the world, in this context its effect is likely minimal due to the high groundwater flow rates from the eastern highlands through to the continental shelf. Perturbing the model to account for a drop in borehole water levels since 2000, we find flow pathways within the SGB Basin become longer particularly within inland regions. A 17 m drop in water levels from groundwater extraction is accommodated by increased recharge by 20% from deeper aquifers of the Great Artesian Basin into surface alluvial sediments, in line with isotopic evidence of groundwater mixing. The insights provided from this study will help to inform an integrated management strategy to support the increasing demand for groundwater in eastern Australia and our integrated modelling framework can be extended to other basins worldwide to explore coupled groundwater-thermal dynamics.

## Methods

Temperature advection due to groundwater flow is described by the advection-diffusion equation, which in steady-state is,

3 $$\begin{aligned} \nabla \cdot ( k_T \nabla T ) + H = C {\mathbf {q}} \cdot \nabla T \end{aligned}$$

where $k_T$ is the thermal conductivity, *H* is the volumetric source term, $c_p$ is the specific heat capacity, ${\mathbf {q}}$ is the flow velocity, and $C=\rho _w C_{p_{w}}$ is the product between the density and heat capacity of water. Darcy flux, ${\mathbf {q}}$, may be calculated from the groundwater flow equation, which in steady state is,

4 $$\begin{aligned} \nabla \cdot {\mathbf {q}}&= 0 \end{aligned}$$

5 $$\begin{aligned} \nabla \cdot (k_h \nabla h )&= 0 \end{aligned}$$

where *h* is the hydraulic head and $k_h$ is the hydraulic conductivity. The coupling is implemented by first solving Eq. (5) and substituting the velocity, ${\mathbf {q}}$, into Eq. (3) to solve for temperature. Since $\nabla T$ changes with every solution, Eq. (3) is non-linear and requires several iterations to converge to a final temperature solution. We implement this model in *Underworld*, a finite element particle-in-cell software framework for geodynamics^18^. The numerical model outputs can be downloaded from https://cloudstor.aarnet.edu.au/plus/s/kNwa6dN6nXxXs55 and viewed in open-source visualisation software such as *Paraview*.

### Boundary conditions

Groundwater recharge and discharge are driven by changes in hydraulic head. The hydraulic head prescribed to the top boundary surface is set to the height of the water Table^45^. In practise, the water table closely resembles a smoother version of the topography without high amplitude hills or mountains (Fig. 1b) and hugs the water level observed in boreholes within shallow (< 200 m deep) unconfined aquifers from the NGIS database. The data were fitted using a weighted spline using the *Stripy* Python package^46^.

The thermal boundary conditions include a constant temperature set to the top boundary ($T_0 = 18$ $^\circ$C), which corresponds to the annual mean surface temperature. The side walls are assigned zero flux and the bottom temperature boundary, $T_1$, is an unknown variable that we invert from borehole temperature data within our Bayesian optimisation scheme described below.

### Temperature-dependent thermal conductivity

Thermal conductivity is dependent on temperature using an Arrhenius law^47^,

6 $$\begin{aligned} k_T(T) = k_{T_0} \left( \frac{T_0}{T} \right) ^a \end{aligned}$$

where $T_0$ is the surface temperature (usually close to 298 K), $k_{T_0}$ is the thermal conductivity at the surface, and $a = 0.33$ for a dominantly silicate composition or 0.25 for mafic rocks. A list of thermal conductivity values relevant to major lithologies in the Sydney–Gunnedah–Bowen Basin are summarised in Table 1.

### Depth-dependent hydraulic conductivity

As rocks become more compressed with depth, their permeability decreases and fluid flow is impeded. The reduction in hydraulic conductivity is generally proportional to depth, *d*^48,49^,

7 $$\begin{aligned} k_h(d) = k_{h_0} \left( 1 - \frac{d}{58 + 1.02 d} \right) ^3 \end{aligned}$$

where $k_{h_0}$ is the ground surface hydraulic conductivity that may vary considerably depending on the rock type. For instance, sediments like sandstone may have a hydraulic conductivity that is 3–5 magnitudes higher than volcanic rocks. A list of hydraulic conductivity values relevant to major lithologies in the Sydney–Gunnedah–Bowen Basin are summarised in Table 1.

### Depth-dependent porosity

The Darcy flux, ${\mathbf {q}}$, is not equivalent to the fluid velocity, ${\mathbf {v}}$. Instead, the velocity at which fluid is flowing through the pores is related to the porosity, $\varphi$,

8 $$\begin{aligned} {\mathbf {v}} = \frac{{\mathbf {q}}}{\varphi } \end{aligned}$$

where $0< \varphi < 1$. The pore space within rocks decrease as they become more compressed with depth. We apply an empirical porosity–depth relationship to our simulations^50^,

9 $$\begin{aligned} \varphi (z) = \frac{\varphi _0}{(1+mz)^n} \end{aligned}$$

where $\varphi _0$ is the porosity at ground surface ($z=0$), and *m* and *n* are fitting constants which are 0.071 and 5.989, respectively for continental crust.

### Formulation of the inverse problem

The inverse solution is given by the *a posteriori* probability function, $P({\mathbf {m}}|{\mathbf {d}})$, which is proportional to the product of the likelihood function, $P({\mathbf {d}}|{\mathbf {m}})$, and the *a priori* probability $P({\mathbf {m}})$.

10 $$\begin{aligned} P({\mathbf {m}}|{\mathbf {d}}) \propto P({\mathbf {d}}|{\mathbf {m}}) \cdot P({\mathbf {m}}) \end{aligned}$$

The likelihood is the probability of reproducing the data ${\mathbf {d}}$ given a particular model ${\mathbf {m}}$, and the *a priori* probability is prior knowledge about the model before assimilating the data. In this case, the model parameters correspond to the lower temperature boundary condition $T_1$ and material properties ($k_T$, $k_h$, *H*) assigned to discreet lithologies in the Sydney–Gunnedah–Bowen Basin; the data corresponds to temperature gradients collected from boreholes, $\Delta T_d$, and groundwater recharge rates estimated from chloride mass balance methods, $q_d$. Therefore, $P({\mathbf {m}}|{\mathbf {d}}) = P(k_T, k_h, H, T_1 | \Delta T_d, q_d)$. The priors for the lithologies are taken from Table 1 and the lower boundary condition is a uniform distribution between 100–1000 $^{\circ }$C. The posterior probability can be evaluated through an objective function, $S({\mathbf {m}})$, which compares the misfit between data and prior information,

11 $$\begin{aligned} P({\mathbf {m}}|{\mathbf {d}}) = A \, \exp (-S({\mathbf {m}})) \end{aligned}$$

where *A* is a constant. The maximum *a posterori* (MAP) estimate is obtained by minimising the $\ell _2$-norm objective function if the priors and data are both uncorrelated,

12 $$\begin{aligned} S({\mathbf {m}}) = \frac{1}{2} \sum _i \frac{|g^i({\mathbf {m}}) - \mathrm {d}^i |^2}{(\sigma _{\mathrm {d}}^i)^2} + \frac{1}{2} \sum _{j} \frac{\vert \mathrm {m}^j - \mathrm {m}^j_p \vert ^2}{(\sigma ^j_p)^2} \end{aligned}$$

where *g* is the forward operator, which is the prediction of observations from the model, ${\mathbf {m}}$. Here, this is the computation of steady-state temperature augmented by the groundwater flow field from which we compare the misfit between temperature profiles from our model and those measured in boreholes.

### Optimisation scheme

We use the *differential evolution* algorithm for finding the global minimum of $S({\mathbf {m}})$^34^. This resides within the family of genetic algorithms that traverse a probability space much more efficiently than traditional Markov-Chain Monte Carlo (MCMC) techniques such as Metropolis Hastings. In differential evolution, multiple starting points (${\mathbf {m}}_0$) are initialised from a Latin hypercube and their local probability space is explored using a MCMC sampler. After a specified number of iterations, multiple minima are culled to a subset of promising candidates, which are further refined using gradient-based optimisation. For this last step, convergence to a local minimum is reached when either $|{\mathbf {m}}_n - {\mathbf {m}}_{n-1}| < 10^{-12}$ or $|S({\mathbf {m}}_n) - S({\mathbf {m}}_{n-1})| < 10^{-12}$. The differential evolution algorithm will return the global minimum of $S({\mathbf {m}})$ provided there is a sufficient number of seed points and MCMC iterations to sample the posterior.

## Supplementary Information

Supplementary Information 1.

Supplementary Information 2.

Supplementary Information 3.

Supplementary Information 4.
